# Intravenous administration of xenogenic adipose-derived mesenchymal stem cells (ADMSC) and ADMSC-derived exosomes markedly reduced brain infarct volume and preserved neurological function in rat after acute ischemic stroke

**DOI:** 10.18632/oncotarget.12902

**Published:** 2016-10-25

**Authors:** Kuan-Hung Chen, Chih-Hung Chen, Christopher Glenn Wallace, Chun-Man Yuen, Gour-Shenq Kao, Yi-Ling Chen, Pei-Lin Shao, Yung-Lung Chen, Han-Tan Chai, Kun-Chen Lin, Chu-Feng Liu, Hsueh-Wen Chang, Mel S. Lee, Hon-Kan Yip

**Affiliations:** ^1^ Department of Anesthesiology, Kaohsiung Chang Gung Memorial Hospital and Chang Gung University College of Medicine, Kaohsiung, Taiwan; ^2^ Department of Internal Medicine, Division of General Medicine, Kaohsiung Chang Gung Memorial Hospital and Chang Gung University College of Medicine, Kaohsiung, Taiwan; ^3^ Department of Plastic Surgery, University Hospital of South Manchester, Manchester, UK; ^4^ Department of Surgery, Division of Neurosurgery, Kaohsiung Chang Gung Memorial Hospital and Chang Gung University College of Medicine, Kaohsiung, Taiwan; ^5^ Department of Internal Medicine, Division of Cardiology, Kaohsiung Chang Gung Memorial Hospital and Chang Gung University College of Medicine, Kaohsiung, Taiwan; ^6^ Department of Nursing, Asia University, Taichung, Taiwan; ^7^ Department of Emergency Medicine, Kaohsiung Chang Gung Memorial Hospital and Chang Gung University College of Medicine, Kaohsiung, Taiwan; ^8^ Department of Biological Sciences, National Sun Yat-Sen University, Kaohsiung, Taiwan; ^9^ Department of Orthopedics, Kaohsiung Chang Gung Memorial Hospital and Chang Gung University College of Medicine, Kaohsiung, Taiwan; ^10^ Institute for Translational Research in Biomedicine, Kaohsiung Chang Gung Memorial Hospital and Chang Gung University College of Medicine, Kaohsiung, Taiwan; ^11^ Center for Shockwave Medicine and Tissue Engineering, Kaohsiung Chang Gung Memorial Hospital and Chang Gung University College of Medicine, Kaohsiung, Taiwan; ^12^ Department of Medical Research, China Medical University Hospital, China Medical University, Taichung, Taiwan

**Keywords:** xenogenic adipose-derived mesenchymal stem cell, exosomes, ischemic stroke, brain infarct size, neurological function, Pathology Section

## Abstract

We tested the hypothesis that combined xenogenic (from mini-pig) adipose-derived mesenchymal stem cell (ADMSC) and ADMSC-derived exosome therapy could reduce brain-infarct zone (BIZ) and enhance neurological recovery in rat after acute ischemic stroke (AIS) induced by 50-min left middle cerebral artery occlusion. Adult-male Sprague-Dawley rats (*n* = 60) were divided equally into group 1 (sham-control), group 2 (AIS), group 3 [AIS-ADMSC (1.2×10^6^ cells)], group 4 [AIS-exosome (100μg)], and group 5 (AIS-exosome-ADMSC). All therapies were provided intravenously at 3h after AIS procedure. BIZ determined by histopathology (by day-60) and brain MRI (by day-28) were highest in group 2, lowest in group 1, higher in groups 3 and 4 than in group 5, but they showed no difference between groups 3 and 4 (all *p* < 0.0001). By day-28, sensorimotor functional results exhibited an opposite pattern to BIZ among the five groups (*p* < 0.005). Protein expressions of inflammatory (inducible nitric oxide synthase/tumor necrosis factor-α/nuclear factor-κB/interleukin-1β/matrix metalloproteinase-9/plasminogen activator inhibitor-1/RANTES), oxidative-stress (NOX-1/NOX-2/oxidized protein), apoptotic (caspase-3/ Poly-ADP-ribose polymerase), and fibrotic (Smad3/transforming growth factor-β) biomarkers, and cellular expressions of brain-damaged (γ-H2AX+/ XRCC1-CD90+/p53BP1-CD90+), inflammatory (CD11+/CD68+/glial fibrillary acid protein+) and brain-edema (aquaporin-4+) markers showed a similar pattern of BIZ among the groups (all *n* < 0.0001). In conclusion, xenogenic ADMSC/ADMSC-derived exosome therapy was safe and offered the additional benefit of reducing BIZ and improving neurological function in rat AIS.

## INTRODUCTION

Acute ischemic stroke (AIS) is the leading cause of long-term disability and the second cause of death worldwide [1–4]. Currently, a universally accepted effective and safe management strategy for patients with AIS remains undefined [5–11]. Thrombolysis with tissue plasminogen activator (tPA) and endovascular intracranial treatment are two emerging treatments for AIS with promising results in specific patient groups; however, strict enrollment criteria and contraindications restrict their scope in clinical practice [12–18]. Furthermore, tPA appears to be associated with a relatively high incidence of intracranial bleeding complications [12–17] and poor patency rate in large-vessel occlusion, and yet endovascular intracranial treatment is only suitable for patients with large-vessel occlusion [18]. Accordingly, the majority of AIS patients still lack a treatment that is both effective and safe. An alternative treatment needs to be developed for AIS patients, particularly for those who are not candidates for thrombolysis or endovascular intracranial treatment.

Plentiful data have demonstrated that stem cell therapies were effective and promising for many diseases refractory to traditional management strategies [18–21]. Of these stem cells, adipose-derived mesenchymal stem cells (ADMSCs) induce angiogenesis, leading to improved ischemia-related organ dysfunction, and have anti-inflammatory and immunomodulatory capacity for attenuating IR-induced organ dysfunction [22–25]. Autologous stem cells were utilized in most of these studies [18–25]; however, allogenic stem cells may also be suitable. Investigators often emphasize that allogenic stem cells are immune privileged and safe for recipients, but this is not universally accepted. Perhaps, a new innovation is required to resolve this issue. Exosomes contain distinct subsets of microRNAs depending upon the cell type from which they are secreted [26]. Several experimental studies have demonstrated that the MSC-derived exosomes have distinctive features of (1) angiogenesis, (2) immunomodulation, and (3) a paracrine effect that improves organ function following injury in preclinical studies [25, 27–31]. Recently, our study [25] showed that ADMSCs and ADMSC-derived exosome therapy significantly protected kidney from ischemia-reperfusion injury. Based on the aforementioned issues, this study tested the therapeutic impact of xenogenic (from mini-pig) ADMSCs and ADMSC-derived exosomes on brain anatomical and neurological functional recoveries in rat after AIS. We also clarified whether xenogenic MSCs exhibit immune privilege like allogenic MSCs reportedly do.

## RESULTS

### H&E staining for examination of the five major organs of brain, heart, lung, liver and kidney by day 21 after ADMSC/ADMSC-derived exosome treatment in healthy animals (Figure 1)

As expected, H&E microscopy revealed no abnormal histopathological findings in these five organs. IF microscopy also did not illustrate any ADMS/ADMSC-derived exosomes present in these organs. Our findings highlighted that exogenic AMDMS/ADMSC-derived exosomes administered to rodent animals should maintain immune privilege without immune reaction and without harm to the healthy animals.

**Figure 1:**
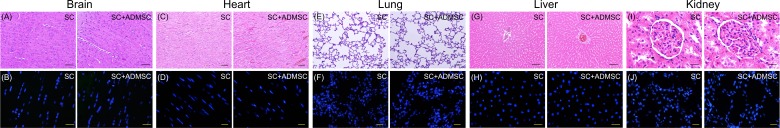
Microscopic findings of five major organs by day 21 after ADMSC/ADMSC-derived exosome treatment in healthy animals (*n* = 4) **A.** Illustrating H.E. stain of brain tissue showing no any lesion/tumorigenesis in the brain area. **B.** Illustrating the immunofluorescent (IF) microscopic finding of brain tissue showing no any dye-stained ADMSC+ cells in the brain area. **C.** Illustrating H.E. stain of left ventricular myocardium (LVM) showing no any lesion/tumorigenesis in LVM. **D.** Illustrating the IF microscopic finding of LVM showing no any dye-stained ADMSC+ cells in the myocardium. **E.** Illustrating H.E. stain of lung tissue showing no any lesion/tumorigenesis in lung parenchyma. **F.** Illustrating the IF microscopic finding of lung tissue showing no any dye-stained ADMSC+ cells in the parenchyma. **G.** Illustrating H.E. stain of liver organ showing no any lesion/tumorigenesis in liver parenchyma. **H.** Illustrating the IF microscopic finding of liver organ showing no any dye-stained ADMSC+ cells in the liver parenchyma. **I.** Illustrating H.E. stain of kidney showing no any lesion/tumorigenesis in kidney parenchyma. **J.** Illustrating the IF microscopic finding of kidney showing no any dye-stained ADMSC+ cells in the kidney parenchyma. ADMSC = adipose-derived mesenchymal stem cell.

### Xenogenic ADMSC/ADMSC-derived exosomes transfusion limited brain infarct size and enhanced recovery of neurological functional without tumorigenesis (Figures 2 and 3)

**Figure 2:**
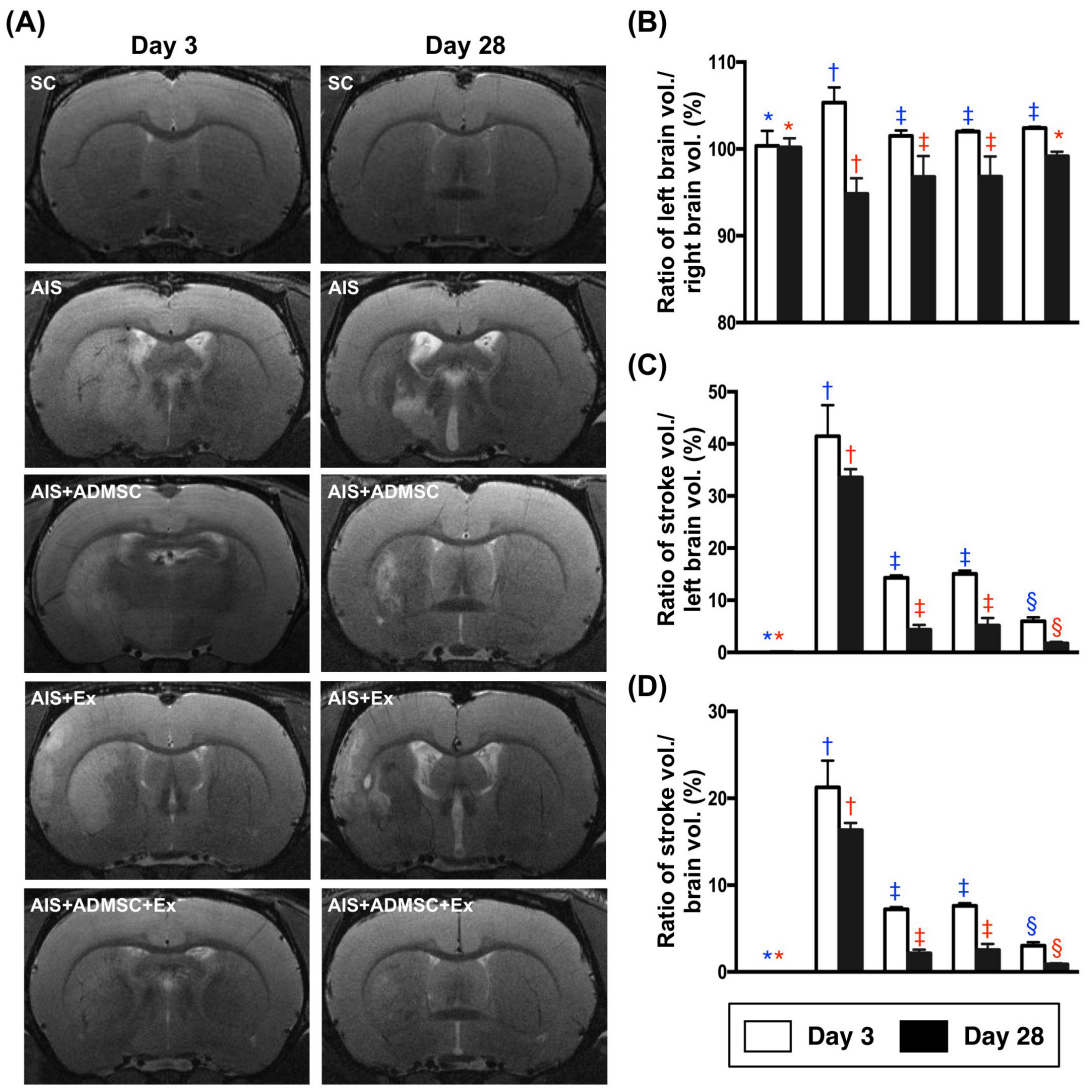
Brain magnetic resonance imaging (MRI) finings in animals by days 3 and 28 after acute ischemic stroke (*n* = 6) **A.** Illustrating brain MRI findings of brain infarction zone (white color) by days 3 and 28 after AIS. **B.** Ratio of left brain volume to right brain volume, by day 3: * *vs*. other groups with different symbols (†, ‡), *p* < 0.001; by day 28, * *vs*. other groups with different symbols (†, ‡). **C.** Ratio of left brain infarct volume to left brain volume (i.e. left hemisphere) x 100%, by day 3: * *vs*. other groups with different symbols (†, ‡, §), *p* < 0.0001; by day 28: * *vs*. other groups with different symbols (†, ‡, §), *p* < 0.0001. **D.** Ratio of left brain infarct volume to total brain volume, by day 3: * *vs*. other groups with different symbols (†, ‡, §), *p* < 0.0001; by day 28: * *vs*. other groups with different symbols (†, ‡, §), *p* < 0.0001. All statistical analyses were performed by one-way ANOVA, followed by Bonferroni multiple comparison post hoc test. Symbols (*, †, ‡, §) indicate significance (at 0.05 level). SC = sham control; AIS = acute ischemic stroke; ADMSC = adipose-derived mesenchymal stem cell; Ex = exosome.

By day 3 after AIS, the brain MRI findings exhibited that the ratio of left brain volume (LBV) to total brain volume (TBV) was significantly higher in AIS only, than in SC, AIS-ADMSC, AIS-Ex and AIS-ADMSC-Ex, and significantly higher in groups AIS-ADMSC, AIS-Ex and AIS-ADMSC-Ex than in SC, but no difference was found among AIS-ADMSC, AIS-Ex and AIS-ADMSC-Ex. Additionally, by day 28 after AIS, the ratio of LBV/TBV was significantly lower in AIS only than in AIS-ADMSC, AIS-Ex and AIS-ADMSC-Ex, and significantly lower in AIS-ADMSC and AIS-Ex than in SC and AIS-ADMSC-Ex, but it showed no difference between AIS-ADMSC and AIS-Ex or between SC and AIS-ADMSC-Ex (Figure 2). These findings suggest that ADMSC-exosome treated attenuated brain swelling in acute stage (i.e., at day 3 after AIS) and reduced the brain shrinkage due to the infarction in recovery stage (i.e., by day 28 after AIS).

**Figure 3:**
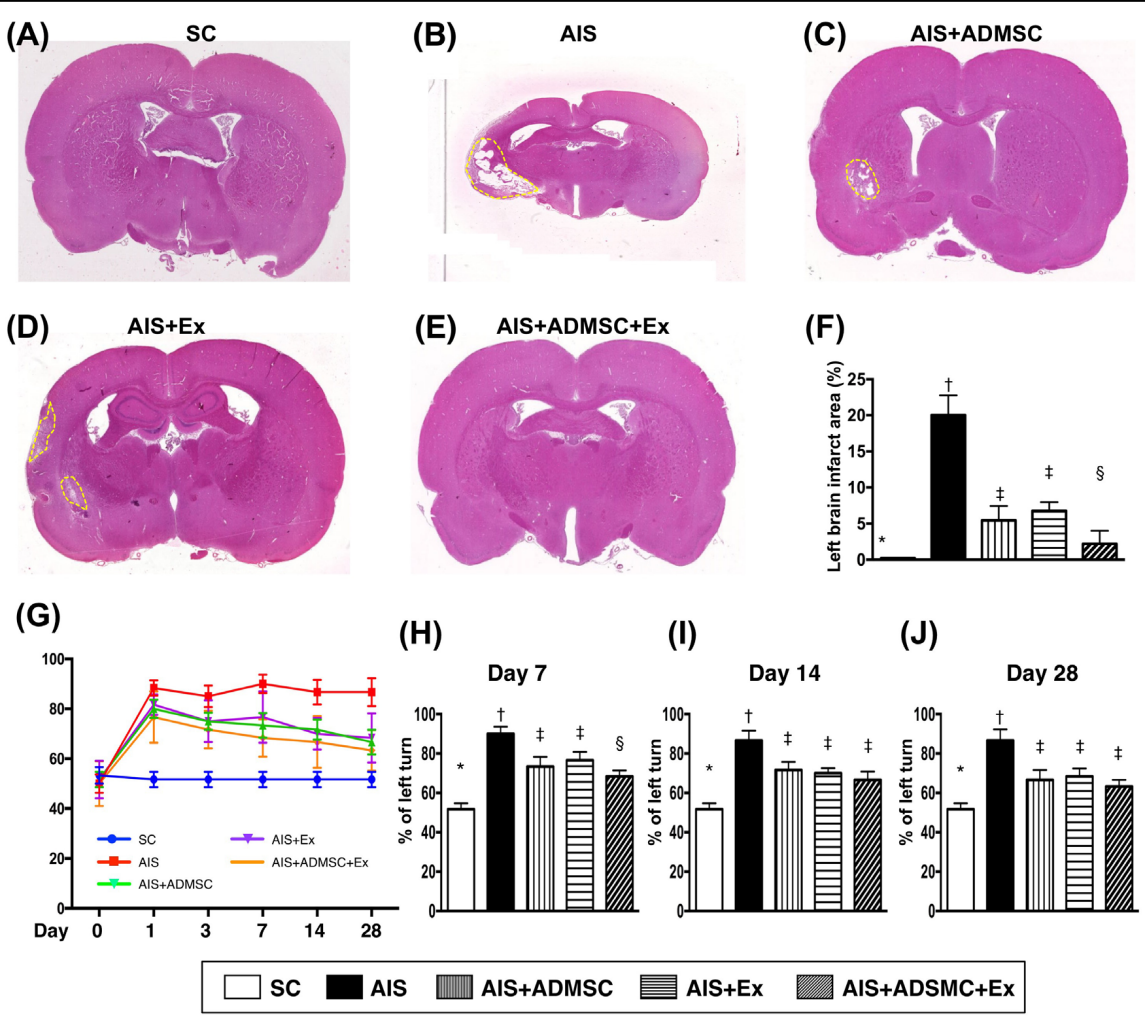
Pathological finding of brain infarct area (BIA) on day 60 and time courses of neurological status after acute ischemic stroke (*n* = 6) **A.** to **E.** Illustrating the H.E. staining (100x) of whole brain cross section for identification of BIA (the yellow dotted line indicated the boundary of BIA). **F.** Statistical analysis of summated (five cross section in each animal) BIA, * *vs*. other groups with different symbols (†, ‡, §), *p* < 0.0001. **G.** Corner test showing the attainment of a steady state of neurological functional impairment from days 1 to 28 following AIS among groups 2 to 5. Significant improvement in neurological function was found apparently in groups 3 to 5 as compared with group 2 by day 7 after AIS. Further notable improvement by day 14 and more further notable improvement by 28 after AIS were observed in groups 3, 4 and 5 but not in group 2. **H.** Statistical analysis by day 7, * *vs*. other groups with different symbols (†, ‡, §), *p* < 0.01. **I.** Statistical analysis by day 14, * *vs*. other groups with different symbols (†, ‡), *p* < 0.001. **J.** Statistical analysis by day 14, * *vs*. other groups with different symbols (†, ‡), *p* < 0.0001. All statistical analyses were performed by one-way ANOVA, followed by Bonferroni multiple comparison post hoc test. Symbols (*, †, ‡) indicate significance (at 0.05 level). SC = sham control; AIS = acute ischemic stroke; ADMSC = adipose-derived mesenchymal stem cell; Ex = exosome.

By day 3 after AIS, the ratio of left brain infarct volume to total left brain volume (LIV/TLBV) and the ratio of LIV/TBV were lowest in SC and highest in AIS only, and significantly lower in AIS-ADMSC-Ex than in AIS-ADMSC and AIS-Ex, but they showed no difference between AIS-ADMSC and AIS-Ex (Figure 2). Additionally, by day 28, the ratios of LIV/TLBV and LIV/TBV exhibited an identical pattern of day 3 among the five groups (Figure 3).

By day 60, the left brain infarct area (i.e., identified by H.E. staining and calculated by summations of five whole brain cross sections in each animal) showed an identical pattern of LIV by 28 after AIS among the five groups (Figure 3). Importantly, no tumorigenesis was evident on brain MRI or pathology (i.e., H.E., stain) in ADMS/ADMSC-derived exosome-treated animals (i.e., in AIS-ADMSC, AIS-Ex and AIS-ADMSC-Ex).

Corner test (Figure 3) demonstrated the attainment of a steady state of neurological functional impairment on day 3 following AIS among AIS-ADMSC, AIS-Ex and AIS-ADMSC-Ex. Additionally, significant improvement in neurological function became apparent in AIS-ADMSC, AIS-Ex and AIS-ADMSC-Ex but not in AIS only compared with SC on day 14. Further substantial improvements in AIS-ADMSC and AIS-Ex and more substantial improvements in AIS-ADMSC-Ex were noted on day 28 after AIS.

### Protein expression of inflammatory and oxidative stress biomarkers in brain infarct zone by day 60 after AIS (Figures 4 and 5)

The protein expressions of MMP-9, IL-1β, TNFα, RANTES, PAI-1, NF-κB and iNOS, seven indicators of inflammation, were highest in AIS only, lowest in SC, significantly higher in AIS-ADMSC and AIS-Ex than in AIS-ADMSC-Ex, and significantly higher in AIS-Ex than in AIS-ADMSC. This identical pattern was found among the five groups for protein expression of NOX-1, NOX-2 and oxidized protein, three indicators of oxidative stress.

**Figure 4:**
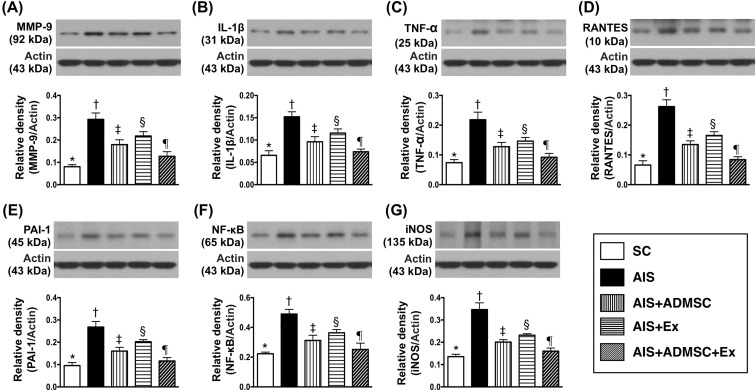
Protein expression of inflammatory biomarkers in brain infarct region by day 60 after AIS (*n* = 6) **A.** Protein expression of matrix metalloproteinase (MM9-9), * *vs*. other groups with different symbols (†, ‡, §, ¶), *p* < 0.0001. **B.** Protein expression of interleukin (IL)-1β, * *vs*. other groups with different symbols (†, ‡, §, ¶), *p* < 0.0001. **C.** Protein expression of tumor necrosis factor (TNF)-α, * *vs*. other groups with different symbols (†, ‡, §, ¶), *p* < 0.0001. **D.** Protein expression of RANTES, * *vs*. other groups with different symbols (†, ‡, §, ¶), *p* < 0.0001. **E.** Protein expression of plasminogen activator inhibitor (PAI)-1, * *vs*. other groups with different symbols (†, ‡, §, ¶), *p* < 0.0001. **F.** Protein expression of nuclear factor (NF)- κB, * *vs*. other groups with different symbols (†, ‡, §, ¶), *p* < 0.0001. **G.** Protein expression of inducible nitric oxide synthase (iNOS), * *vs*. other groups with different symbols (†, ‡, §, ¶), *p* < 0.0001. All statistical analyses were performed by one-way ANOVA, followed by Bonferroni multiple comparison post hoc test. Symbols (*, †, ‡, §, ¶) indicate significance (at 0.05 level). SC = sham control; AIS = acute ischemic stroke; ADMSC = adipose-derived mesenchymal stem cell; Ex = exosome.

**Figure 5:**
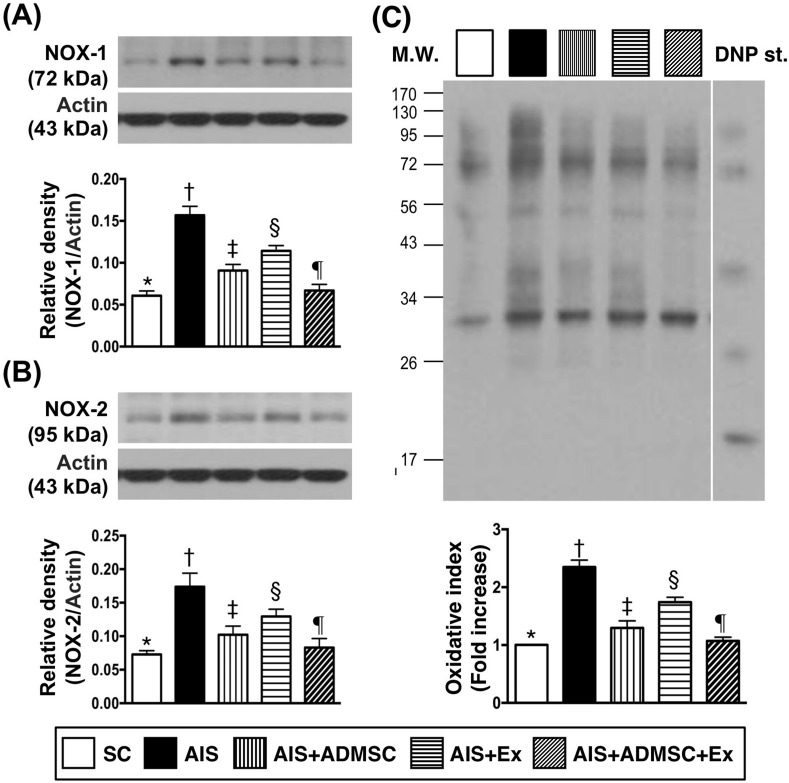
Protein expression of oxidative stress biomarkers in brain infarct region by day 60 after AIS (*n* = 6) **A.** Protein expression of NOX-1, * *vs*. other groups with different symbols (†, ‡, §, ¶), *p* < 0.0001. **B.** Protein expression of NOX-2, * *vs*. other groups with different symbols (†, ‡, §, ¶), *p* < 0.0001. **C.** Oxidized protein expression, * *vs*. other groups with different symbols (†, ‡, §, ¶), *p* < 0.0001. (Note: left and right lanes shown on the upper panel represent protein molecular weight marker and control oxidized molecular protein standard, respectively). M.W = molecular weight; DNP = 1-3 dinitrophenylhydrazone. All statistical analyses were performed by one-way ANOVA, followed by Bonferroni multiple comparison post hoc test. Symbols (*, †, ‡, §, ¶) indicate significance (at 0.05 level). SC = sham control; AIS = acute ischemic stroke; ADMSC = adipose-derived mesenchymal stem cell; Ex = exosome.

### Protein expressions of apoptotic, fibrotic and anti-fibrotic biomarkers in brain infarct zone by day 60 after AIS (Figure 6)

The protein expressions of cleaved caspase 3 and cleaved PARP, two indicators of apoptosis, were highest in AIS only, lowest in SC, significantly higher in AIS-ADMSC and AIS-Ex than in AIS-ADMSC-Ex, and significantly higher in AIS-Ex than in AIS-ADMSC. The protein expressions of Smad3 and TGF-β, two indices of fibrosis, exhibited an identical pattern of apoptosis among the five groups. The protein expressions of Smad1/5 and BMP-2, two indicators of anti-fibrosis, displayed an opposite pattern of apoptosis among the five groups.

**Figure 6:**
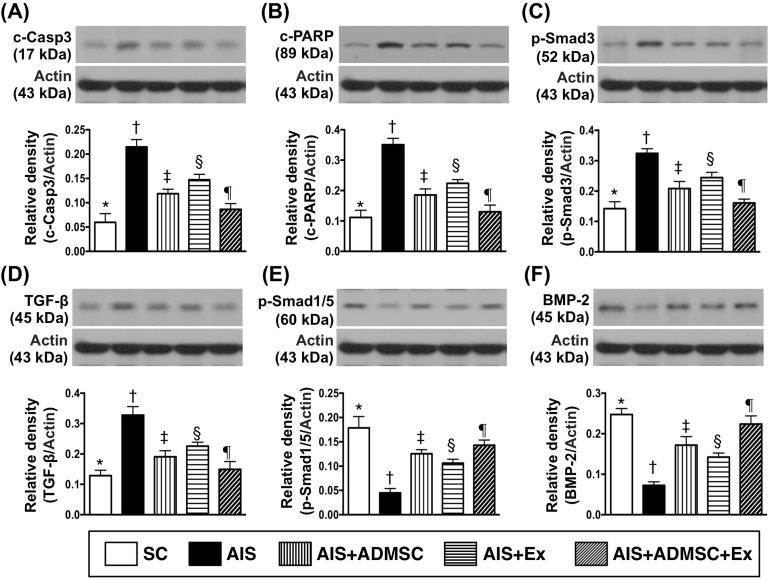
Protein expressions of apoptotic, fibrotic and anti-fibrotic biomarkers in brain infarct zone by day 60 after AIS (*n* = 6) **A.** Protein expression of cleaved caspase 3 (c-Casp 3), * *vs*. other groups with different symbols (†, ‡, §, ¶), *p* < 0.0001. **B.** Protein expression of cleaved poly (ADP-ribose) polymerase (c-PARP), * *vs*. other groups with different symbols (†, ‡, §, ¶), *p* < 0.0001. **C.** Protein expression of phosphorylated (p)-Smad3, * *vs*. other groups with different symbols (†, ‡, §, ¶), *p* < 0.0001. **D.** Protein expression of transforming growth factor (TGF)-β, * *vs*. other groups with different symbols (†, ‡, §, ¶), *p* < 0.0001. **E.** Protein expression of p-Smad1/5, * *vs*. other groups with different symbols (†, ‡, §, ¶), *p* < 0.0001. **F.** Protein expression of bone morphogenetic protein (BMP)-2, * *vs*. other groups with different symbols (†, ‡, §, ¶), *p* < 0.0001. All statistical analyses were performed by one-way ANOVA, followed by Bonferroni multiple comparison post hoc test. Symbols (*, †, ‡, §, ¶) indicate significance (at 0.05 level). SC = sham control; AIS = acute ischemic stroke; ADMSC = adipose-derived mesenchymal stem cell; Ex = exosome.

### Protein expressions of DNA-damage, mitochondrial-damage and angiogenesis markers in brain infarct zone by day 60 after AIS (Figure 7)

The protein expressions of γ-H2AX, a DNA-damage marker, and cytosolic cytochrome C, a mitochondrial-damage marker, was highest in AIS only, lowest in SC, significantly higher in AIS-ADMSC and AIS-Ex than in AIS-ADMSC-Ex, and significantly higher in AIS-Ex than in AIS-ADMSC. On the other hand, protein expression of mitochondrial cytochrome C, an indicator of mitochondrial integrity, showed an opposite pattern to γ-H2AX among the five groups. Additionally, the protein expressions of CD31 and eNOS, two indicators of endothelial function, exhibited an identical pattern to mitochondrial cytochrome C among the five groups. Furthermore, the protein expressions of VEGF and CXCR4, two indices of angiogenesis, exhibited significantly progressive increases from groups SC to AIS-ADMSC-Ex, suggesting an intrinsic response to ischemic stimulation.

**Figure 7:**
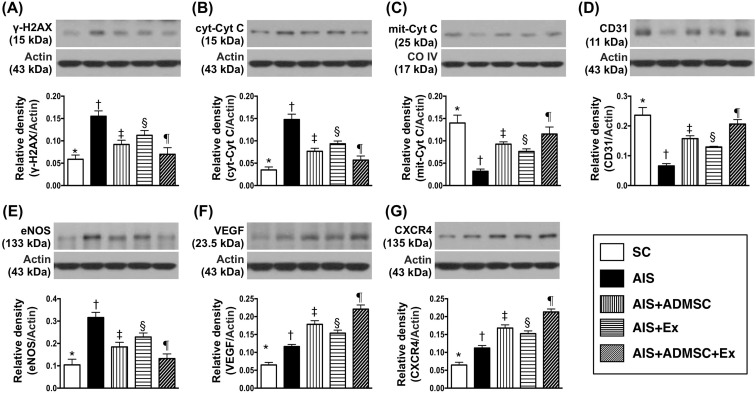
Protein expressions of DNA-damaged and mitochondrial-damaged markers and angiogenesis markers in brain infarct zone by day 60 after AIS (*n* = 6) **A.** Protein expression of γ-H2AX, * *vs*. other groups with different symbols (†, ‡, §, ¶), *p* < 0.0001. **B.** Cytosolic cytochrome C (cyt-Cyt C), * *vs*. other groups with different symbols (†, ‡, §, ¶), *p* < 0.0001. **C.** Protein expression of mitochondria cytochrome C (mit-Cyt C), * *vs*. other groups with different symbols (†, ‡, §, ¶), *p* < 0.0001. **D.** Protein expression of CD31, * *vs*. other groups with different symbols (†, ‡, §, ¶), *p* < 0.0001. **E.** Protein expression of endothelial nitric oxide synthase (eNOS), * *vs*. other groups with different symbols (†, ‡, §, ¶), *p* < 0.0001. **F.** Protein expressions of vascular endothelial growth factor (VEGF), * *vs*. other groups with different symbols (†, ‡, §, ¶), *p* < 0.0001. **G.** Protein expression of CXCR4, * *vs*. other groups with different symbols (†, ‡, §, ¶), *p* < 0.0001. All statistical analyses were performed by one-way ANOVA, followed by Bonferroni multiple comparison post hoc test. Symbols (*, †, ‡, §, ¶) indicate significance (at 0.05 level). SC = sham control; AIS = acute ischemic stroke; ADMSC = adipose-derived mesenchymal stem cell; Ex = exosome.

### Cellular expressions of DNA-damage, inflammatory, and brain-damage biomarkers in brain infarct zone by day 60 after AIS (Figures 8, 9 and 10)

**Figure 8:**
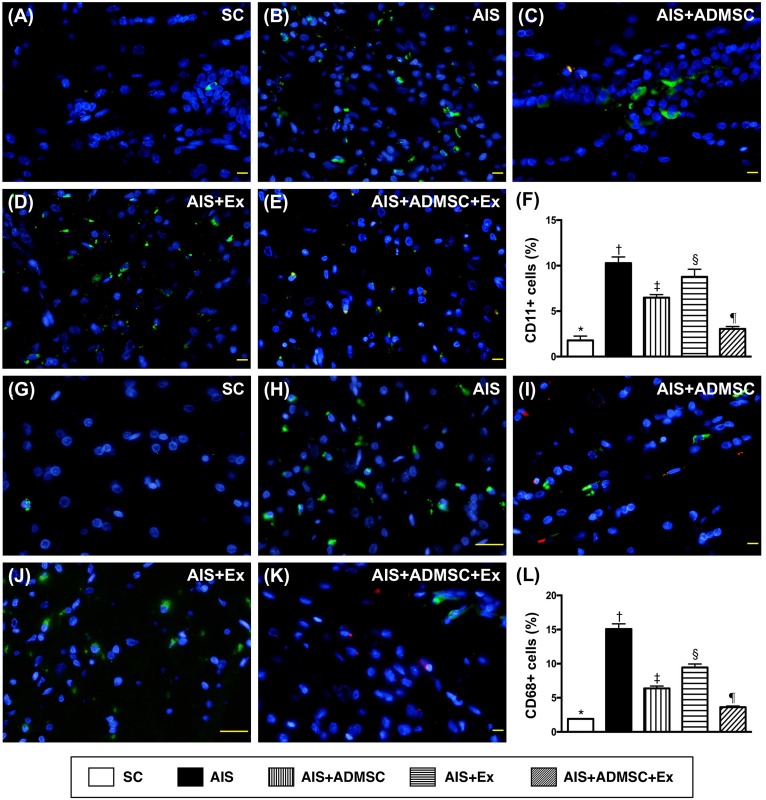
Inflammatory cell expression in brain infarct zone by day 60 after AIS (*n* = 6) **A.** to **E.** Illustrating immunofluorescent (IF) microscopic finding (400x) of CD11+ cells (green color). Red-color indicated Dil-dye stained ADMSC. **F.** Analytical results of number of CD11+ cells, *vs. other groups with different symbols (†, ‡, §, ¶), *p* < 0.0001. **G.** to **K.** Illustrating IF microscopic findings (400x) of CD68+ cells (green color). Red-color indicated Dil-dye stained ADMSC. **L.** Analytical results of number of CD68+ cells, *vs. other groups with different symbols (†, ‡, §, ¶), *p* < 0.0001. Scale bars in right lower corner represent 20μm. All statistical analyses were performed by one-way ANOVA, followed by Bonferroni multiple comparison post hoc test. Symbols (*, †, ‡, §, ¶) indicate significance (at 0.05 level). SC = sham control; AIS = acute ischemic stroke; ADMSC = adipose-derived mesenchymal stem cell; Ex = exosome.

**Figure 9:**
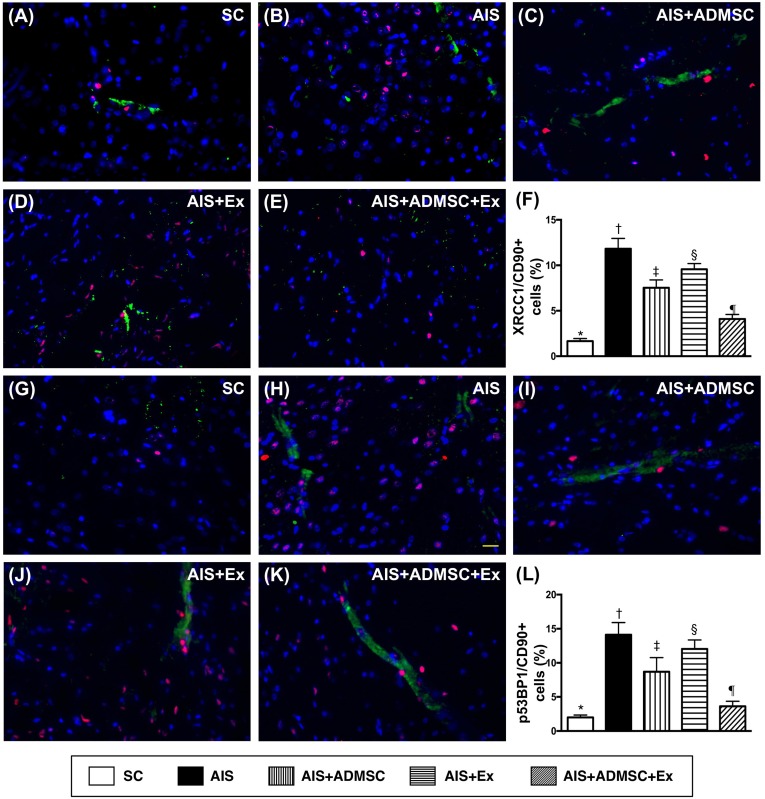
Microscopic findings of double-stranded DNA damaged positively stained cells in brain infarct zone by day 60 after AIS (*n* = 6) **A.** to **E.** Illustrating immunofluorescent (IF) microscopic finding (400x) of XRCC1/CD90+ cells (pink color). **F.** Analytical results of number of XRCC1/CD90+ cells, *vs. other groups with different symbols (†, ‡, §, ¶), *p* < 0.0001. **G.** to **K.** Illustrating IF microscopic findings (400x) of p53BP1/CD90+ cells (pink color). **L.** Analytical results of number of p53BP1/CD90+ cells, *vs. other groups with different symbols (†, ‡, §, ¶), *p* < 0.0001. Scale bars in right lower corner represent 20μm. All statistical analyses were performed by one-way ANOVA, followed by Bonferroni multiple comparison post hoc test. Symbols (*, †, ‡, §, ¶) indicate significance (at 0.05 level). SC = sham control; AIS = acute ischemic stroke; ADMSC = adipose-derived mesenchymal stem cell; Ex = exosome.

**Figure 10:**
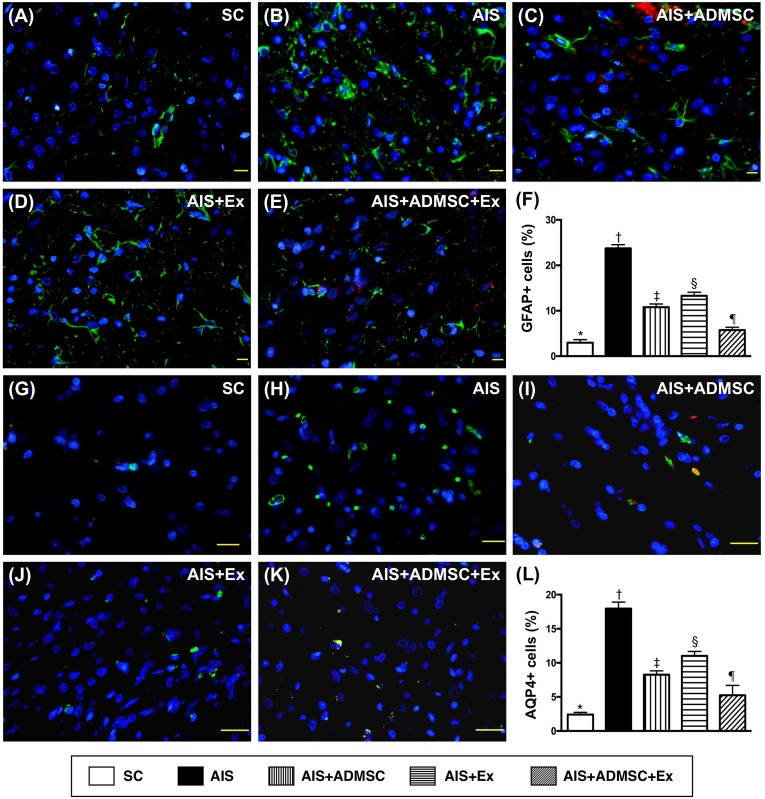
Cellular expressions of GFAP and AQP4 in brain infarct zone by day 60 after AIS (*n* = 6) **A.** to **E.** Immunofluorescent (IF) microscope (400x) identifying glial fibrillary acid protein (GFAP)+ cells (green color). Red-color indicated Dil-dye stained ADMSC. **F.** Analytical results of number of GFAP+ cells, *vs. other groups with different symbols (†, ‡, §, ¶), *p* < 0.0001. **G.** to **K.** IF microscope (400x) identifying aquaporin 4 (AQP4)+ cells (green color). **L.** Analytical results of number of GFAP+ cells, *vs. other groups with different symbols (†, ‡, §, ¶), *p* < 0.0001. Scale bars in right lower corner represent 20μm. All statistical analyses were performed by one-way ANOVA, followed by Bonferroni multiple comparison post hoc test. Symbols (*, †, ‡, §, ¶) indicate significance (at 0.05 level). SC = sham control; AIS = acute ischemic stroke; ADMSC = adipose-derived mesenchymal stem cell; Ex = exosome.

The IF microscopic findings demonstrated that the cellular expressions (Figure 8) of CD11 and CD68, two indicators of inflammation, were highest in AIS only, lowest in SC, significantly higher in AIS-ADMSC and AIS-Ex than in AIS-ADMSC-Ex, and significantly higher in AIS-Ex than in AIS-ADMSC. IF microscopic findings also showed that the cellular expressions (Figure 9) of XRCC1/CD90 and p53BP1/CD90, two indicators of double-stranded DNA damage, exhibited an identical pattern to inflammation among the five groups. Furthermore, the cellular expressions (Figure 10) of GFAP (a specific glial cell marker, indicating inflammation) and AQP4 (an indicator of brain edema) showed an identical pattern to inflammation among the five groups.

### Microscopic findings of angiogenesis and endothelial cellular expressions in brain infarct zone by day 60 after AIS (Figures 11 and 12)

**Figure 11:**
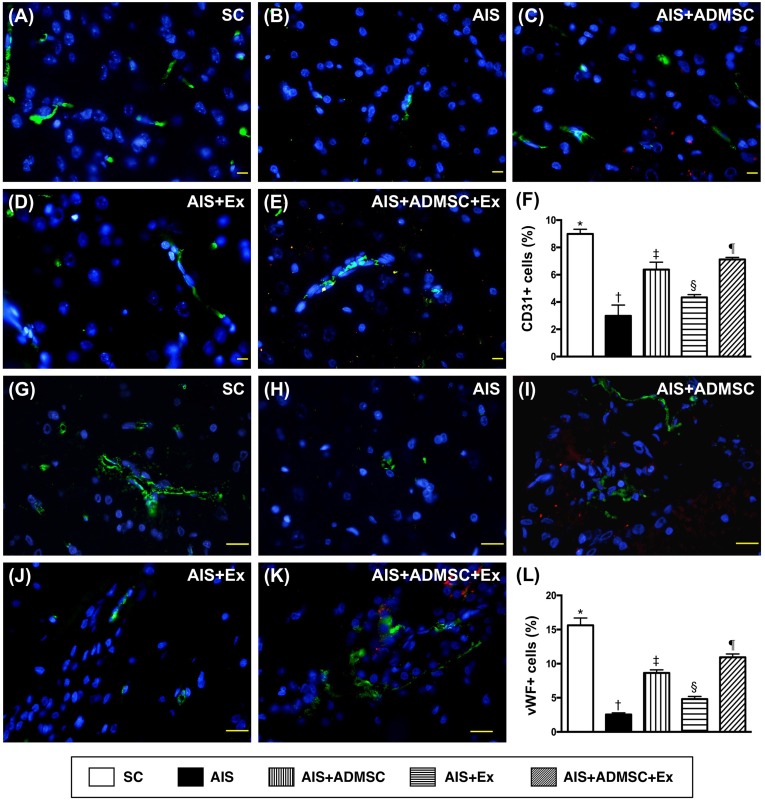
Microscopic findings of expressions of endothelial surface markers in brain infarct zone by day 60 after AIS (*n* = 6) **A.** to **E.** Immunofluorescent (IF) microscopic finding (400x) of CD31+ cells (green color). Red-color indicated Dil-dye stained ADMSC. **F.** Analytical results of number of CD31+ cells, *vs. other groups with different symbols (†, ‡, §, ¶), *p* < 0.0001. **G.** to **K.** IF microscopic finding (400x) of von Willebrand factor (vWF)+ cells (green color). Red-color indicated Dil-dye stained ADMSC. **L.** Analytical results of number of vWF+ cells, *vs. other groups with different symbols (†, ‡, §, ¶), *p* < 0.0001. Scale bars in right lower corner represent 20μm. All statistical analyses were performed by one-way ANOVA, followed by Bonferroni multiple comparison post hoc test. Symbols (*, †, ‡, §, ¶) indicate significance (at 0.05 level). SC = sham control; AIS = acute ischemic stroke; ADMSC = adipose-derived mesenchymal stem cell; Ex = exosome.

**Figure 12:**
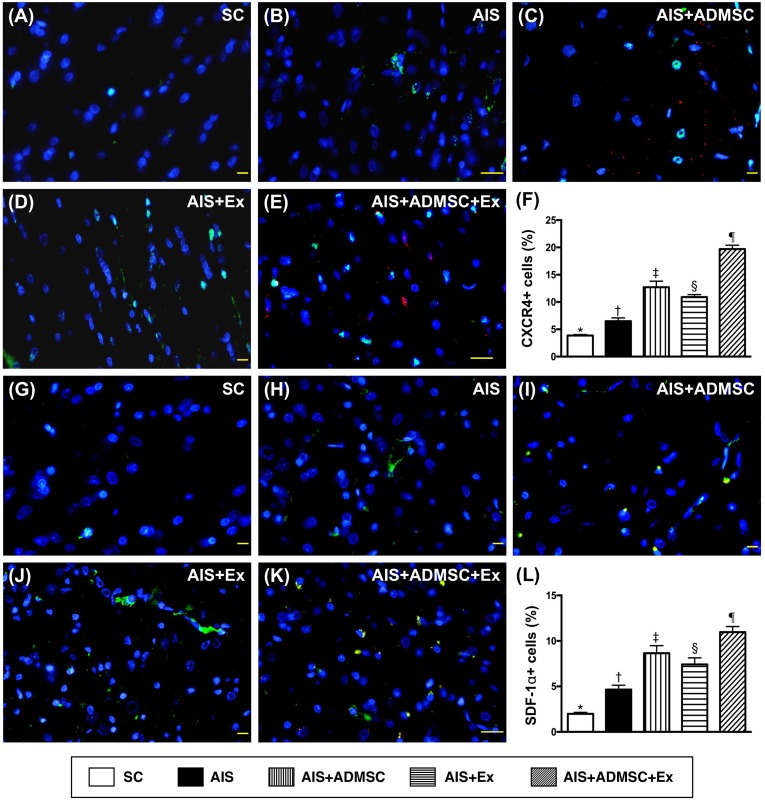
Microscopic findings of angiogenesis expressions in brain infarct zone by day 60 after AIS (*n* = 6) **A.** to **E.** Immunofluorescent (IF) microscopic finding (400x) of CXCR4+ cells (green color). Red-color indicated Dil-dye stained ADMSC. **F.** Analytical results of number of CXCR4+ cells, *vs. other groups with different symbols (†, ‡, §, ¶), *p* < 0.0001. **G.** to **K.** IF microscopic finding (400x) of stromal cell derived factor (SDF)-1+ cells (green color). Red-color indicated Dil-dye stained ADMSC. **L.** Analytical results of number of SDF-1α+ cells, *vs. other groups with different symbols (†, ‡, §, ¶), *p* < 0.0001. Scale bars in right lower corner represent 20μm. All statistical analyses were performed by one-way ANOVA, followed by Bonferroni multiple comparison post hoc test. Symbols (*, †, ‡, §, ¶) indicate significance (at 0.05 level). SC = sham control; AIS = acute ischemic stroke; ADMSC = adipose-derived mesenchymal stem cell; Ex = exosome.

The cellular expressions of CD31 and vWF (Figure 11), two indicators of endothelial function integrity, were highest in SC, lowest in AIS only, significantly higher in AIS-ADMSC-Ex than in AIS-ADMSC and AIS-Ex, and significantly higher in AIS-ADMSC than in AIS-Ex. Additionally, the cellular expressions of CXCR4 and SDF-1α (Figure 12), two indicators of angiogenesis, showed significant and progressive increases from groups SC to AIS-ADMSC-Ex, implicating an intrinsic response to ischemic stimulation.

### Small vessel density and γ-H2AX+ cell expressions in brain infarct zone (BIZ) by day 60 after AIS (Figure 13)

The number of small vessels in BIZ, an indicator of angiogenesis, was highest in SC, lowest in AIS only, significantly higher in AIS-ADMSC-Ex than in AIS-ADMSC and AIS-Ex, and significantly higher in AIS-ADMSC than in AIS-Ex. The γ-H2AX+ cell expression, another DNA-damage biomarker, was highest in AIS only, lowest in SC, significantly higher in AIS-ADMSC and AIS-Ex than in AIS-ADMSC-Ex, and significantly higher in AIS-Ex than in AIS-ADMSC.

**Figure 13:**
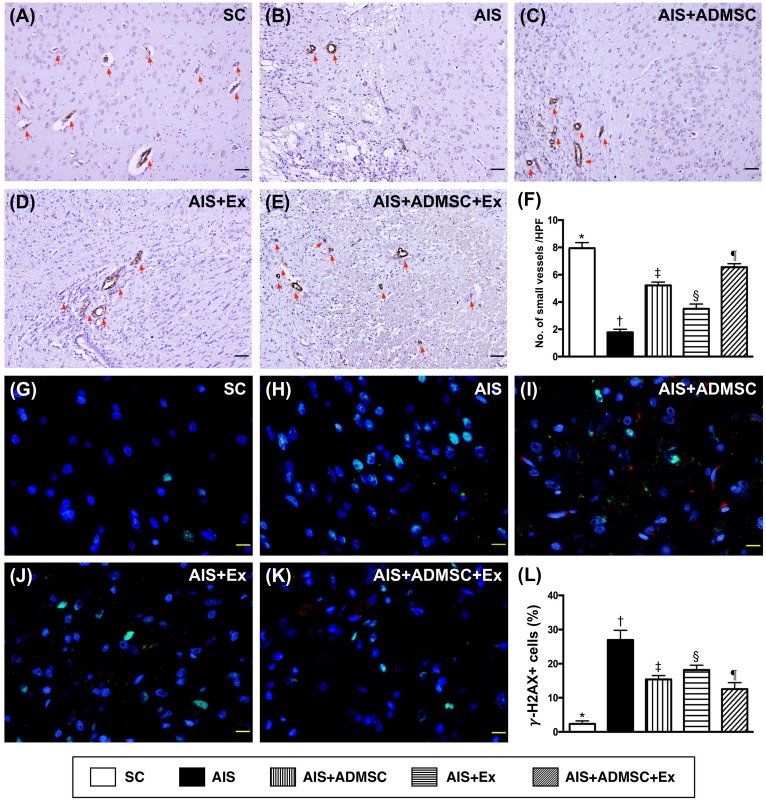
Small vessel density and γ-H2AX+ cell expressions in brain infarct zone by day 60 after AIS (*n* = 6) **A.** to **E.** Microscopic (100x) finding of immunohistochemical staining (i.e., α-smooth muscle actin positive stain) for identifying the number of small vessels (≤ 25 μM) (red arrows). **F.** Results of statistical analysis, *vs. other groups with different symbols (†, ‡, §, ¶), *p* < 0.0001. The scale bars in right lower corner represent 100 μm. HPF = high-power field. **G.** to **K.** Immunofluorescent microscopic finding (400x) of γ-H2AX+ cell + cells (green color). Red-color indicated Dil-dye stained ADMSC. **L.** Analytical results of number of γ-H2AX+ cell cells, *vs. other groups with different symbols (†, ‡, §, ¶), *p* < 0.0001. Scale bars in right lower corner represent 20μm. All statistical analyses were performed by one-way ANOVA, followed by Bonferroni multiple comparison post hoc test. Symbols (*, †, ‡, §, ¶) indicate significance (at 0.05 level). SC = sham control; AIS = acute ischemic stroke; ADMSC = adipose-derived mesenchymal stem cell; Ex = exosome.

## DISCUSSION

Our recent study showed that allogenic ADMSC/ADMSC-derived exosome therapy significantly protected kidney architecture, glomerular ultrastructure and renal function from acute kidney ischemia-reperfusion injury in rodent [25]. In addition, this study proved that allogenic ADMSC/ADMSC-derived exosomes were immune privileged in rodent [25]. An essential finding in the present study was that immune reaction was not identified and major organs (brain, heart, lung, liver and kidney) were not injured by xenogenic ADMSC/ADMSC-derived exosome treatment. Additionally, the most important findings in the present study were that, compared with AIS animals, the BIZ (i.e., by MRI and histopathological findings) was reduced and neurological function (i.e., by sensorimotor functional test) was improved in AIS animals treated by xenogenic ADMSC or ADMSC-derived exosomes. Also, combined therapy with the two regimens offered additional benefit over either one therapy alone for reducing brain infarct volume and enhancing recovery of neurological function. Our findings, in addition to extending the findings of our recent study [25], highlighted two important issues: (1) xenogenic ADMSC/ADMSC-derived exosome has the property of immune privilege; (2) these xenogenic cell-based therapies safely and effectively protect the brain from AIS in rodent.

It has long been known that innate immune reactions are quickly activated following tissue/organ ischemic injury and necrosis, which, in turn, elicit the complement cascade, inflammation and the generation of oxidative stress and reactive oxygen species (ROS) [19, 32–35]. These cellular-molecular perturbations, finally, directly participate in further tissue/organ damage after ischemic injury [2–5, 19, 21–25, 32, 35–39]. On the other hand, numerous studies have previously shown that MSCs, especially ADMSCs, have powerful anti-inflammatory and immunomodulatory properties that can attenuate ischemia-reperfusion or sepsis induced organ damage [21–25, 39]. One important finding in the present study was that the protein and cellular levels of inflammation in the BIZ were substantially higher in AIS animals compared to control animals. Additionally, oxidative stress biomarkers were higher in AIS animals than in control animals. Accordingly, our findings corroborate those of previous studies [19, 22, 32–35, 39]. Importantly, inflammation, the generation of ROS and oxidative stress were significantly suppressed by ADMSC-derived exosome therapy, more significantly suppressed by ADMSC therapy, and most significantly suppressed by their combined therapy in AIS animals. Interestingly, our recent study [31] showed that ADMSC-derived exosome therapy markedly suppressed the growth of hepatocellular carcinoma mainly through upregulating the natural killer T-cell response. Additionally, our other studies [21–24, 32] have recently shown that allogenic and autologous ADMSCs have immunomodulatory and anti-inflammatory properties, and suppress the production of oxidative stress in the setting of ischemia-reperfusion injury. Furthermore, other studies [40, 41] have exhibited that human-derived endothelial progenitor cell (EPC) therapy effectively improved ischemia-related organ dysfunction in rat (i.e. xenogenic EPC therapy). Accordingly, our findings strengthen those of our recent studies [21–24, 31, 32, 40, 41] and extend the indications for ADMSC/ADMSC-derived exosomes, in that they protect organs from ischemic attack (i.e., from allogenic to xenogenic levels).

A strong correlation between inflammation/oxidative stress and cellular apoptosis/death, fibrosis of organ and tissues, as well as DNA/mitochondria damage in the setting of ischemic injury has been keenly investigated by previous studies [19, 22–25, 32, 35, 37–39]. These molecular-cellular perturbations, in turn, resulted in organ damage and dysfunction [19, 22–25, 32, 35, 37–39]. A principal finding in the present study was that, when compared with the control group, these aforementioned molecular-cellular perturbations were substantially increased in AIS animals. In this way, our findings could, in addition to being comparable with the findings of several previous studies [19, 22–25, 32, 35, 37–39], explain why the BIZ was notably increased and the neurological function was significantly impaired in animals after AIS. Importantly, both BIZ and neurological function in AIS animals were significantly reversed after receiving ADMSC-derived exosome treatment and more significantly reversed after ADMSC treatment. Of particular importance, combined therapy with ADMSC/ADMSC-derived exosomes was superior to either therapy alone at inhibiting the BIZ and improving the recovery of neurological function in animals after AIS.

Another important finding in the present study was that the angiogenesis biomarkers (i.e., CD31+ and vWF+ cell, protein expression of eNOS, VEGF, CXCR4 and number of small vessels in BIZ) were significantly higher in AIS animals with xenogenic ADMSC/ADMSC-derived exosome treatment than in those without. Intriguingly, our recent studies [22, 39] have also displayed that ADMSC treatment for rat with acute ischemia-reperfusion injury markedly enhanced the generation of angiogenesis biomarkers in the region of ischemia. Accordingly, our findings could, in addition to corroborating the findings of our previous studies [22, 39], explain the improvement of neurological function and reduction of brain infarct volume, at least in part, through restoring blood flow in the ischemic region.

### Study limitation

This study has limitations. First, this study only considered the management of AIS and therefore did not provide any information regarding the impact of xenogenic ADMSC/ADMSC-derived exosome therapy on chronic IS. Second, this study did not test the optimal dosage of ADMSCs or ADMSC-derived exosomes on improving the neurological outcome. Accordingly, we remain uncertain whether ADMSC therapy or ADMSC-derived exosome therapy was superior at improving the outcomes in rat after AIS, when both dosage regimens have been optimized.

In conclusion, the present study provided evidence that xenogenic cell-based therapy was safe and efficacious for improving outcomes in rat after AIS.

## MATERIALS AND METHODS

### Animals and ethics statement

All animal experimental protocols and procedures were approved by the Institute of Animal Care and Use Committee at Kaohsiung Chang Gung Memorial Hospital (Affidavit of Approval of Animal Use Protocol No. 2011053001) and performed in accordance with the Guide for the Care and Use of Laboratory Animals [The Eighth Edition of the Guide for the Care and Use of Laboratory Animals (NRC 2011)].

Animals were housed in an Association for Assessment and Accreditation of Laboratory Animal Care International (AAALAC)-approved animal facility in our hospital with controlled temperature and light cycles (24°C and 12/12 light cycle).

### Animal model of acute ischemic stroke and corner test for assessment of neurological function

The protocol and procedure for the experimental rodent AIS model have previously been described [32, 36]. In detail, adult male Sprague-Dawley rats, weighing 350-375 g (Charles River Technology, BioLASCO Taiwan Co., Ltd., Taiwan), were utilized in the current study. Each animal was anesthetized by 2% inhalational isoflurane in a supine position on a warming pad (37°C). After exposure of the left common carotid artery (LCCA) through a transverse neck incision, a small arteriotomy was performed on the LCCA through which a 0.28 mm diameter nylon monofilament was carefully advanced into the distal left internal carotid artery for occlusion of the left middle cerebral artery; this caused brain ischemia and infarction of its supplied area. The nylon monofilament was removed 50 min after occlusion, followed by closure of the muscle and skin in layers. The rats were then cared for in a portable animal intensive care unit (ThermoCare^®^) with food and water for 24 hours.

The sensorimotor functional test (Corner test) was conducted for each rat at baseline and on days 1, 3, 14 and 28 after AIS induction as we recently described [32, 36]. Briefly, the rat was allowed to walk through a tunnel and then into a corner, the angle of which was 60 degrees. To exit the corner, the rat could turn either left or right. The results were recorded by a technician who was blind to the study design. This test was repeated 10 to 15 times with at least 30 seconds between each trial. We recorded the number of right and left turns from 10 successful trials for each animal and used the results for statistical analysis.

### Animal grouping and the treatment protocol

Sixty rats were equally categorized into sham control (SC) (by incision of the neck skin and dissection of the LCCA only), AIS only, AIS + ADMSC [(1.2×10^6^ cells) by intravenous administration at 3 h after AIS procedure], AIS + exosomes [(AIS-Ex) by intravenous administration of 100 μg at 3 h after AIS procedure] and AIS-Ex-ADMSC. The utilized dosages of Ex and ADMSC were based on our recent investigations [21–25, 32].

### Procedure and protocol for isolating and culturing ADMSCs, exosome protein quantification, characterization, and preparation

The procedure and protocol for ADMSC isolation and culture have been described in details in our recent reports [25, 31]. Briefly, abdominal adipose tissue from eight mini-pigs (about 16-18kg) was carefully dissected and excised. ADMSCs were automatically isolated using the KISO™ System (Keai Bioresearch, BC, Canada). For purification, the harvested cells were cultured in a 100 mm diameter dish with MEM-α culture medium containing 10% FBS for 14 days. By this time plentiful ADMSCs were obtained in the culture plate; they were collected to treat AIS animals. Surface markers for ADMSCs were identified by flow cytometric analyses as reported in our previous studies [25, 31].

The procedure to isolate exosomes from the culture medium has been detailed in our recent report [25, 31]. Briefly, exosomes were isolated from the culture medium of ADMSCs and were pooled for electron microscopic assessment, protein separation and characterization, and western blot analysis. The proteins in Dulbecco's Modified Eagle Medium (DMEM) (Gibco) supplemented with 10% serum before and after cell culture were separated by sodium dodecyl sulfate-polyacrylamide gel electrophoresis (SDS-PAGE). The exosomes in DMEM were purified and the proteins in different exosome fractions (1 μg, 2 μg, 10 μg, and 50 μg) were separated by SDS-PAGE. The gel was stained with Coomassie blue for analysis. The following primary antibodies were used: mouse monoclonal anti-CD63 (Santa Cruz Biotechnology), rabbit polyclonal anti-tumor susceptibility gene-101 (TSG101) (Abcam) and anti-β-catenin (Abcam).

### Histopathological findings for identification of ADMSC/ADMSC-derived exosomes in brain, heart liver and lung tissues

To elucidate whether treatment with xenogenic ADMSC or ADMSC-derived exosomes (stained by CM-Dil dye for tracking the ADMSC/exosome) would be harmful to the normal organs/tissues, an additional eight healthy animals (4 per group) were utilized to receive these two different therapies intravenously. By day 21, all animals were euthanized and specimens were collected. Microscopic investigations (Figure 1) demonstrated that no any abnormal findings were identified in any individual organ/tissue.

### Procedure and protocol of brain magnetic resonance imaging (MRI) study

The procedure and protocol for brain magnetic resonance imaging (MRI) study were based on our recent report [36]. MRI was performed at days 3 and 28 after AIS induction. Briefly, during MRI measurements, mice were anesthetized by 3% inhalational isoflurane with room air and placed in an MRI-compatible holder (Biospec 94/20, Bruker, Ettingen, Germany). Rectal temperature and respiration were monitored throughout the procedure to ensure normal physiological conditions were being maintained. MRI data were collected using a Varian 9.4T animal scanner (Biospec 94/20, Bruker, Ettingen, Germany) with a rat surface array. The MRI protocol consisted of 40 T2-weighted images. Forty continuous slice locations were imaged with a field-of-view of 30 mm x 30 mm, an acquisition matrix dimension of 256 × 256 and slice thickness of 0.5 mm. The repetition time (TR) and echo time (TE) for each fast spin-echo volume were 4200 ms and 30 ms, respectively. Custom software, ImageJ (1.43i, NIH, USA), was used to process the region of interest (ROI). Planimetric measurements of images from MRI T2 were performed to calculate the stroke volumes of cortex.

### Specimen collection and preparation for individual study

Animals were euthanized by day 60 after the AIS procedure. For examination of protein expression, the brain of each rat (*n* = 6 for each group) was promptly removed, immersed in cold saline, snap-frozen in liquid nitrogen and then stored at −80°C for individual study. For immunofluorescent (IF) and immunohistochemical (IHC) staining studies, the brains of 6 other animals in each group were reperfused with normal saline via the carotid artery, removed, fixed with 4% paraformaldehyde in 1x PBS (pH7.4), and soaked in 20% sucrose in 1x PBS (freshly prepared) until the brain took on a completely sunken appearance. The sucrose was then discarded and the brain soaked in 30% sucrose in 1x PBS (freshly prepared) for 48 h. The infarcted and non-infarcted parts were then collected. Finally, the OCT block (Tissue-Tek, Sakura, Netherlands) was prepared for IHC and IF staining.

### Immunofluorescent (IF) staining of brain specimens

The procedure and protocol of IF and IHC staining were based on our previous reports [36–38]. In detail, frozen sections (4 μm thick) were obtained from the brain infarct zone (BIZ) of each animal, permeated with 0.5% Triton X-100, and incubated with antibodies against glial fibrillary acid protein (GFAP) (1:500, DAKO), aquaporin4 (AQP4) (1:200, Abcam), vascular endothelial growth factor (VEGF) (1:400, Abcam), CD31 (1:100, Abcam), von Willebrand factor (vWF) (1:200, Abcam), CXCR4 (1:100, Abcam), stromal cell-derived factor (SDF)-1α (1:100, Santa Cruz), CD11 (1:200, Abcam), CD68 (1:100, Abcam), and γ-H2AX (1:5000, Abcam), p53BP1/CD90 (1:200, Novus Biologicals/1:100, Abcam), and XRCC1/CD90 (1:200, Abcam/1:100, Abcam) at 4 ^°^C overnight. Alexa Fluor488, Alexa Fluor568, or Alexa Fluor594-conjugated goat anti-mouse or rabbit IgG were used to localize signals. Sections were finally counterstained with DAPI and observed with a fluorescent microscope equipped with epifluorescence (Olympus IX-40).

Three brain sections were analyzed for each rat. For quantification, three randomly selected high-power fields (HPFs; 400x for IF study) were analyzed in each section. The mean number of positively-stained cells per HPF for each animal was then determined by summation of all numbers divided by 9.

### Vessel density in BIZ

The procedure and protocol for identifying small vessels in the BIZ were based on our previous reports [32, 36–38]. In detail, IHC staining of small blood vessels was performed with α-SMA (1:400) as primary antibody at room temperature for 1 hour, followed by washing with PBS thrice. Ten minutes after the addition of anti-mouse-HRP conjugated secondary antibody, the tissue sections were washed with PBS thrice. Then 3,3' diaminobenzidine (DAB) (0.7 gm/tablet) (Sigma) was added, followed by washing with PBS thrice after one minute. Finally, hematoxylin was added as a counter-stain for nuclei, followed by washing twice with PBS after one minute. Three brain sections were analyzed in each rat. For quantification, three randomly selected HPFs (100x) were analyzed in each section. The mean number per HPF for each animal was then determined by summation of all numbers divided by 9.

### Western blot analysis

The procedure and protocol for Western blot analysis were based on our recent reports [19, 21–25, 32, 36]. Briefly, equal amounts (50 mg) of protein extracts were loaded and separated by SDS-PAGE using acrylamide gradients. After electrophoresis, the separated proteins were transferred electrophoretically to a polyvinylidene difluoride membrane (GE, UK). Nonspecific sites were blocked by incubation of the membrane in blocking buffer [5% nonfat dry milk in T-TBS (TBS containing 0.05% Tween 20)] overnight. The membranes were incubated with the indicated primary antibodies [Caspase 3 (1: 1000, Cell Signaling), Poly (ADP-ribose) polymerase (PARP) (1: 1000, Cell Signaling), nuclear factor (NF)-κB (1: 600, Abcam), tumor necrosis factor (TNF)-α (1: 1000, Cell Signaling), interleukin (IL)-1b (1: 1000, Cell Signaling), RANTES (1: 1000, Cell Signaling), matrix metalloproteinase (MMP)-9 (1: 1000, Abcam), CD31 (1: 1000, Abcam), cytochrome (cyt) C (1: 1000, BD), Phosphorylated (p)-Smad1/5 (1: 1000, Cell Signaling), bone morphogenetic protein (BMP)-2 (1: 500, Abcam), p-Smad3 (1: 1000, Cell Signaling), transforming growth factor (TGF)-b (1:500, Abcam), NOX-1 (1: 1500, Sigma), NOX-2 (1: 750, Sigma), plasminogen activator inhibitor (PAI)-1 (1: 1000, Abcam), von Willebrand factor (vWF) (1: 1000, Abcam), vascular endothelial growth factor (VEGF) (1: 1000, Abcam), inducible nitric oxide synthase (iNOS) (1: 500, Abcam), endothelial nitric oxide synthase (eNOS) (1: 1000, Abcam), γ-H2AX (1: 1000, Cell Signaling), and Actin (1: 1000, Millipore)] for 1 hour at room temperature. Horseradish peroxidase-conjugated anti-rabbit immunoglobulin IgG (1:2000, Cell Signaling, Danvers, MA, USA) was used as a secondary antibody for one-hour incubation at room temperature. The washing procedure was repeated eight times within one hour. Immunoreactive bands were visualized by enhanced chemiluminescence (ECL; Amersham Biosciences, Amersham, UK) and exposed to Biomax L film (Kodak, Rochester, NY, USA). For the purpose of quantification, ECL signals were digitized using Labwork software (UVP, Waltham, MA, USA).

### Assessment of oxidative stress

The procedure and protocol for evaluating the protein expression of oxidative stress have been described in detail in our previous reports [19, 21–25, 32, 36]. The Oxyblot Oxidized Protein Detection Kit was purchased from Chemicon, Billerica, MA, USA (S7150). DNPH derivatization was carried out on 6 μg of protein for 15 minutes according to the manufacturer's instructions. One-dimensional electrophoresis was carried out on 12% SDS/polyacrylamide gel after DNPH derivatization. Proteins were transferred to nitrocellulose membranes that were then incubated in the primary antibody solution (anti-DNP 1: 150) for 2 hours, followed by incubation in secondary antibody solution (1:300) for 1 hour at room temperature. The washing procedure was repeated eight times within 40 minutes. Immunoreactive bands were visualized by ECL (Amersham Biosciences, Amersham, UK), which was then exposed to Biomax L film (Kodak, Rochester, NY, USA). For quantification, ECL signals were digitized using Labwork software (UVP, Waltham, MA, USA). For oxyblot protein analysis, a standard control was loaded on each gel.

### Statistical analysis

Quantitative data are expressed as means ± SD. Statistical analysis was adequately performed by ANOVA, followed by Bonferroni multiple-comparison post hoc test. SAS statistical software for Windows version 8.2 (SAS institute, Cary, NC) was utilized. A P value of less than 0.05 was considered statistically significant.
